# Emerging Risks in Food: Probiotic Enterococci Pose a Threat to Public Health through the Food Chain

**DOI:** 10.3390/foods10112846

**Published:** 2021-11-18

**Authors:** Wenjiao Xu, Yuwen Fang, Qiao Hu, Kui Zhu

**Affiliations:** National Center for Veterinary Drug Safety Evaluation, College of Veterinary Medicine, China Agricultural University, Beijing 100193, China; xwjvet@163.com (W.X.); fangyuwen@cau.edu.cn (Y.F.); qiaohu4-c@my.cityu.edu.hk (Q.H.)

**Keywords:** probiotic, enterococci, toxicity, antimicrobial resistance

## Abstract

Probiotics have been associated with clinical infections, toxicity, and antimicrobial resistance transfer, raising public concerns. Probiotic enterococci are emerging food risks as opportunistic pathogens, yet little attention has been paid to them. Herein, we collected 88 enterococcal isolates from probiotic products used for humans, companion animals, livestock, and aquaculture. Results showed that all 88 probiotic enterococcal isolates harbored diverse virulence genes, multiple antimicrobial resistance genes, and mobile genetic elements. Notably, 77 isolates were highly resistant to gentamicin. Representative enterococcal isolates exerted toxic activities in both in vitro and in vivo models. Collectively, our findings suggest that probiotic enterococci may be harmful to hosts and pose a potential threat to public health.

## 1. Introduction

Probiotics can confer a health benefit on the host when administered in adequate amounts [1], such as activating host immunity [2], improving the function of the intestinal barrier [3], and inhibiting bacterial pathogens [4,5]. The occurrence of antimicrobial resistance in some countries has risen to above 90% [6]. This challenge promotes the need for probiotic products as antimicrobial alternatives. However, probiotics have also been associated with adverse effects. For instance, probiotics have led to infections in certain patient groups world widely [7], delayed mucosal microbiome reconstitution and host transcriptome recovery [8], and caused bacteremia within intensive care unit (ICU) patients [9].

Furthermore, probiotics bear the risks of spreading virulence genes (VGs) and antimicrobial resistance genes (ARGs) [10]. Our previous studies have reported that toxins and mobile ARGs were found in probiotic *Bacillus* strains [11,12]. Notably, a toxin-positive probiotic *Bacillus cereus* strain has transferred into groundwater and then to a nearby fish farm [13]. Therefore, risky probiotics could be a reservoir for transferrable VGs and ARGs, and increase the emergence and dissemination of potential harmful factors.

Enterococci are the natural colonizers of the gastrointestinal tract in humans and animals. They have been used as starter cultures for over a century and have been applied in probiotic products for recent decades. Nevertheless, they are also recognized as severe opportunistic pathogens that can acquire and express multi-drug resistance and numerous virulence traits [14]. The possession of ARGs and VGs are important components for probiotic safety assessment [15]. At present, many studies have found safety concerns for enterococci as probiotics [7,16,17]. Remarkably, the vancomycin resistance gene *vanA*, is transferees from an *Enterococcus* spp. strain to *L. acidophilus* in an animal model, suggesting that ARGs of enterococci can be disseminated in vivo [18]. Furthermore, a previous report has demonstrated that probiotics could be transmitted from farms to humans [13]. Therefore, probiotic enterococci with transferrable VGs and ARGs are hazardous to the host and public health.

As the suitability of enterococci is growingly questioned, the European Food Safety Authority determined that enterococci did not meet “Qualified Presumption of Safety” status [19], and endorsed the pathogenesis of *Enterococcus faecalis* (*E. faecalis*) and *Enterococcus faecium* (*E. faecium*). However, *E. faecium* is still used as feed supplements in the USA, termed as direct-fed microorganisms. Although *E. faecium* and *E. faecalis* have been prohibited in food materials since 1 July 2019 in Taiwan, China [20], they are still widely used in probiotic products for various application purposes in Chinese mainland.

A few studies have evaluated the safety of human- and animal-use probiotic products [12,21], yet the current literature is not well equipped to illuminate the effects of probiotic enterococci on the host. Herein, our study aimed to comprehensively evaluate the probiotic enterococci with a focus on the capability of producing toxicity and carrying ARGs, thus assessing their potential risks for the host and public health.

## 2. Materials and Methods

### 2.1. Isolation and Identification of Bacteria

A commercially available probiotic product (1 g) was added to 9 mL phosphate-buffered saline (PBS) and vortexed thoroughly. Serial 10-fold dilutions were then made in PBS, and a 100 μL volume of each dilution was inoculated onto Bile Esculin Azide (BEA) agar plates. *Enterococcus* spp. were identified by BEA agar plates. The species-specific identification was first performed by matrix-assisted laser desorption ionization-time of flight mass spectrometry (AXIMA Performance, Shimadzu, Japan). Then, the isolates were identified by 16S rRNA sequence analysis as a previous study described [22]. After the dual identification of chromogenic selective medium and 16S rRNA sequence analysis, genomic DNA of all probiotic enterococci isolates was extracted by the Hipure Bacterial DNA Kit (Magen, Guangzhou, China) and subsequently determined by whole-genome sequencing.

### 2.2. Bioinformatics Analysis

A total of 88 *E. faecium* and *E. faecalis* isolates were selected for whole-genome sequencing (WGS). Multilocus sequence typing (MLST) results were analyzed using MLST Version 2 (https://github.com/tseemann/mlst) on 18 December 2020. The VGs, ARGs, and mobile genetic elements (MGEs) of isolates were identified using ABRicate (https://github.com/tseemann/ABRicate) on 12 March 2021 and the National Center for Biotechnology Information (NCBI) databases on 12 March 2021. Phylogenetic trees for 85 *E. faecium* isolates based on the core genome sequences were structured using Harvest version 1.1.2, with the corresponding characteristics of each isolate visualized using the online tool iTOL version 4. It is possible to search for genes with specified similarity from 70–100% identity, and the best-matching genes are given as output [23]. Possible metabolites were predicted through the perspective of bacterial gene analysis by antiSMASH bacterial version.

### 2.3. Upper Gastrointestinal Transit Tolerance Assay

Simulated gastric juices were prepared by suspending pepsin (Aladdin, Shanghai, China) in sterile saline (0.5% *w/v*) to a final concentration of 2 g/L, and we adjusted the pH to 2.0 with the concentrated HCl using a pH meter (Sartorius, Göttingen, Germany). Simulated small intestinal juices were prepared by suspending pancreatin USP (Sigma, P1750, St. Louis, MO, USA) in sterile saline (0.5% *w/v*) to a final concentration of 1 g/L, and adjusting the pH to 8.0 with sterile 0.1 mol/L NaOH using the pH meter. An aliquot (0.2 mL) of each representative isolate was transferred to a 2.0 mL tube, and then mixed with 0.3 mL of NaCl (0.5% *w/v*) and 1.0 mL of simulated gastric (pH = 2.0) or small intestinal juices (pH = 8.0). The mixture was then vortexed at the maximum setting for 10 s and incubated at 37 °C. When screening gastric and small intestinal transit tolerance, aliquots of 0.1 mL were removed after 0, 1, 2, and 3 h for determination of the total viable count.

### 2.4. Antibacterial Properties

The antibacterial activity of representative isolates was determined by the Oxford cup plate method. Four Oxford cups were placed with equal intervals on brain heart infusion agar (BHA) plates, and then 10^6^ CFU/mL of *S. aureus* ATCC29213, *S. aureus* T144, *E. coli* 25922, and *E. coli* B2 were spread on the surface of the BHA agar of different plates, respectively. After the agar was solidified, Oxford cups were removed and 50 µL of probiotic enterococci suspensions (10^9^ CFU/mL) were injected into the wells. These plates were incubated at 37 °C for 24 h and the zones of inhibition were measured.

### 2.5. Bacteria–Cell Coculture

Enterococci-mediated cytotoxicity was performed on the human intestinal epithelial cells (HIEC cells). For the observation of cell morphology, 1.0 × 10^5^/mL of HIEC cells were coincubated with 1.0 × 10^7^ CFU/mL of representative enterococcal isolates in 6-well plates (Multiplicity of Infection (MOI) = 100), and cultured in DMEM/F12 with 10% FBS for 6 h. Lastly, images were obtained by a microscope (Leica, Wetzlar, Hesse-Darmstadt, Germany).

Rates of cell survival were determined in a colorimetric assay based on the measurement of lactate dehydrogenase (LDH) released from lysed HIEC cells. Briefly, 1.0 × 10^5^/mL of HIEC cells was co-incubated with 1.0 × 10^7^ CFU/mL of enterococcal isolates in 96-well plates (MOI = 100). After a six-hour incubation period in a humidified chamber (37 °C, 5% CO_2_), lysed cells were used to account for spontaneous LDH release activity. The spontaneous remained LDH activity in HIEC cells correlates with cytotoxicity of representative enterococcal isolates, and it was determined using an LDH cytotoxicity assay kit (Beyotime, Shanghai, China) according to the manufacturer’s instructions. The absorbance was measured at 490 nm by a microplate reader (SpectraMax M5, Molecular Devices, San Jose, CA, USA) within one hour.

### 2.6. Confocal Laser Scanning Microscopy

HIEC cells were infected with representative enterococcal isolates for 6 h (MOI = 100). F-actin were stained by GFP (Green) and nuclei were counterstained with DAPI (Blue). Representative enterococcal isolates were stained by rhodamine phalloidin (Red). For static images, stained cellular samples were captured by a Leica SP confocal microscope. 3D images were taken to capture all the X-, Y-, and *Z*-axis sections. Images were analyzed and merged by the LAS AF Lite software (Leica, Wetzlar, Hesse-Darmstadt, Germany).

### 2.7. Assessment of Phenotypic Antimicrobial Resistance

Minimum inhibitory concentrations (MICs) of enterococcal isolates were determined by classical micro-broth dilution method according to the Clinical and Laboratory Standards Institute (CLSI)’s operating directions (CLSI M100S-S26). Antimicrobial drugs used in the assay contained ampicillin (AMP), ciprofloxacin (CIP), florfenicol (FFC), linezolid (LZD), rifampicin (RIF), tetracycline (TET), erythrocin (ERY), vancomycin (VAN), and gentamicin (GEN). *Staphylococcus aureus* ATCC29213 was chosen as a standard control for antimicrobial susceptibility tests.

### 2.8. Galleria Mellonella Survival Assays

*G. mellonella* in the final instar larval stage (300 mg body weight, purchased from Tianjin Huiyude Biotech Company, Tianjin, China) was used in this assay. Two control groups were included: one inoculated with PBS as a control for physical trauma, and the other not injected as a control for general viability. A 10 µL Hamilton syringe was used to inject 10 µL inoculum aliquots (representative enterococcal isolates at 10^7^ CFUs) into the hemocoel of each larva via the penultimate proleg. After the injection, larvae were incubated at 37 °C in plastic containers. Larvae were observed every 6 h (0 h, 6 h, 12 h, 18 h, 24 h, 30 h, 36 h, 42 h), and considered dead when they displayed no movement in response to touch. Ten randomly chosen *G. mellonella* larvae were used per group in all assays.

### 2.9. Mouse Intestinal Infection Model

Thirty-two female ICR (Institute of Cancer Research) mice (8 weeks of age; 28~33 g; Vital River Laboratory Animal Technology Co. Ltd., Beijing, China) were used in this study. All mice were housed in four cages (8 mice per cage) under conventional conditions and had free access to food and water. After adaptation for one week, the mean weight of mice in four cages was 36.32 g, 36.01 g, 36.18 g, and 36.21 g, respectively. The mice were randomly divided into four groups: control group, mice solely treated with PBS; antibiotic supplement group, mice with intragastric administration with 0.1 mL streptomycin (200 mg/mL); Efm65 supplement group, mice with intragastric administration with *E. faecium* 65 (Efm65, 1.0 × 10^9^ CFUs in 200 μL PBS); and antibiotic+Efm65 supplement group, mice with intragastric administration with Efm65 at 24 h post-antibiotic pretreatment. After that, the drinking water was replaced with sterile water.

#### 2.9.1. Bacterial Population

Fecal pellets were collected and resuspended in PBS to count the bacterial population after the treatment of 24 to 96 h. At 96 h, mice in each group were sacrificed and the contents of jejunum, ileum, cecum, and colon were collected to count the bacterial population of the Efm65.

#### 2.9.2. Histological Staining

Colonic tissues in 4% paraformaldehyde solution were embedded in paraffin and then sliced for hematoxylin/eosin (HE) staining. Images were photographed at 100× magnification with a microscope (BX51; Olympus, Tokyo, Japan).

### 2.10. Statistical Analysis

Data in the figures were presented as the mean ± standard error. The statistical analysis in this study was performed by SPSS 23.0 (IBM, Armonk, NY, USA). Differences were statistically analyzed by the unpaired Student’s *t*-test method and comparisons among more than two groups were obtained by one-way analysis of variance (ANOVA). *p* ≤ 0.05 was considered to be significant.

## 3. Results

### 3.1. Characterization and Isolation of Probiotic Enterococcal Isolates

**Figure 1:**
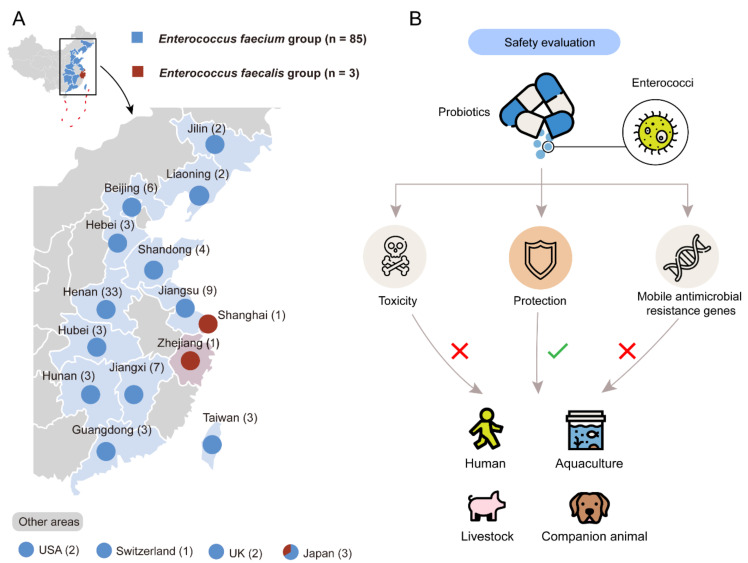
Characterization and evaluation of enterococci from probiotic products. (**A**) Characterization and isolation of enterococci. A total of 88 enterococcal isolates were collected from probiotic products in 12 provinces in China, as well as the USA, UK, Switzerland, and Japan. Numbers in the parentheses indicate the numbers of the enterococci isolates in each place. (**B**) Systematic safety evaluation of probiotic enterococci.

To evaluate the risks of probiotic enterococci, we collected 72 brands of probiotic products containing enterococci from 14 provinces in China, as well as the USA, the UK, Switzerland, and Japan between 2018 and 2020 (Figure 1A). A total of 88 enterococcal isolates were collected, including 85 *E. faecium* isolates and 3 *E. faecium* isolates. These isolates were mostly used for livestock (n = 50), and others were used for companion animals (n = 15), aquaculture and environment (n = 14) and humans (n = 9), respectively (Figure S1). The detailed information of all isolates is demonstrated in Table S1. We randomly chose four enterococcal isolates, which are recovered from products used for humans (*E. faecium* 5, Efm5), companion animals (*E. faecium* 46, Efm46), aquaculture (*E. faecium* 65, Efm65), and livestock (*E. faecium* 81, Efm81), respectively, for the following experiments. The systematical safety assessment of probiotic enterococci is shown in Figure 1B. It provides a standard assessment for commercial probiotic products, thus screening for safe strains.

### 3.2. Representative Probiotic Enterococcal Isolates Survived Steadily under Simulated Gastric and Intestinal Juices

To survive and proliferate in the host, enterococci need to be screened for their tolerance in the upper gastrointestinal tract conditions. The effect of simulated gastric juices (pH = 2.0) on the viability of four representative probiotic enterococcal isolates is presented in Figure S2A. The results showed that all tested isolates retained a similar level of viability during simulated gastric tract transit for 3 h. Three isolates, namely Efm5, Efm46, and Efm81, showed no population reduction, and only one isolate, Efm65, showed a slight reduction (less than one log) of viability. The effect of simulated intestinal juices (pH = 8.0) on the viability of these isolates is presented in Figure S2B. The results showed that all tested isolates had a progressive growth during 3 h of simulated intestinal transit. Therefore, these enterococcal isolates are highly tolerant to the upper gastrointestinal transit, which allows them to persist and proliferate in the host.

### 3.3. Representative Probiotic Enterococcal Isolates Had No Antibacterial Activity on Pathogenic Bacteria

**Figure 2:**
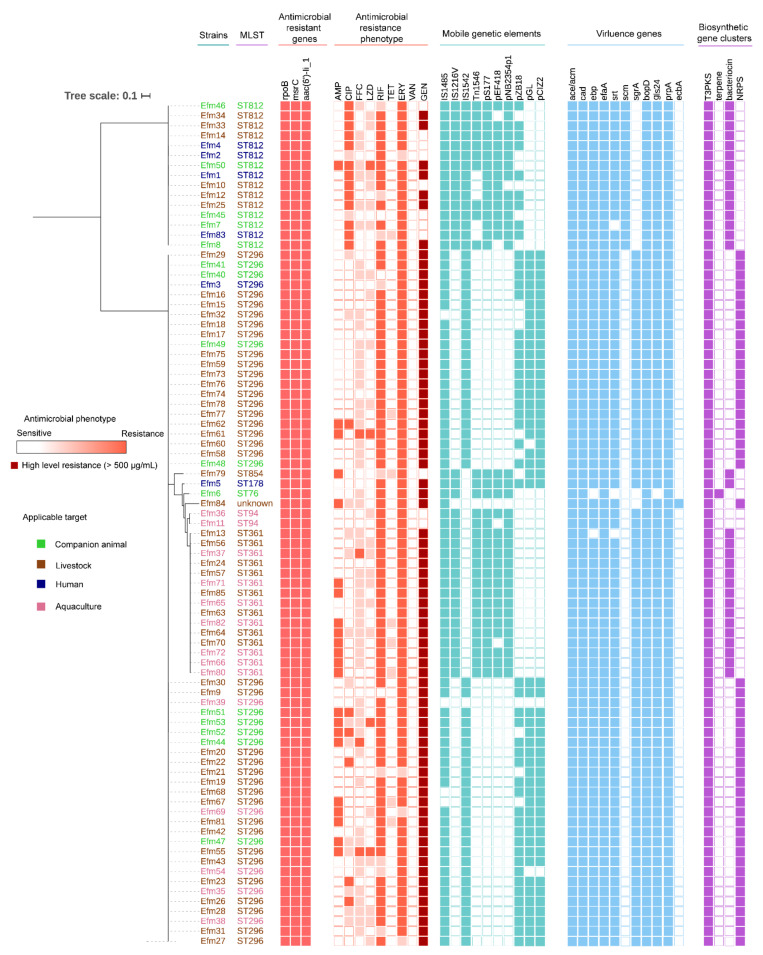
Phylogenetic tree and multilocus sequencing types, antimicrobial resistance genes (ARGs) and phenotype, mobile genetic elements (MGEs), virulence genes (VGs), and biosynthetic gene clusters (BGCs) among enterococcal isolates from probiotic products. The sources of the isolates are differentiated by color. Filled squares are for presence and empty squares are for absence.

Firstly, we predicted possible metabolites of enterococcal isolates through the perspective of bacterial gene analysis by antiSMASH bacterial version. The results of biosynthetic gene clusters including polyketide synthase (T3PKS), terpene, bacteriocin, and non-ribosomal peptide synthase (NRPS) are presented in Figure 2. Nevertheless, none of them had a similar known cluster, indicating that the bacterial genes may be silent. In order to detect the direct antibacterial activity of probiotic enterococci, we performed the Oxford cup plate experiment. The results showed that none of the four representative enterococcal isolates had antibacterial activity.

### 3.4. All Probiotic Enterococcal Isolates Had Antimicrobial Resistance

Worse still, we found antimicrobial resistance traits in these probiotic enterococcal isolates both in genetic and phenotypic analysis. Firstly, ARGs and MGEs were determined by analyzing whole-genome data. In Figure 2, all 85 *E. faecium* isolates carried antimicrobial resistant genes: *msrC*, *rpoB*, and *aac(6′)-Ii*. These antimicrobial-resistant genes corresponded to the resistance to ERY (*msrC*), RIF (*rpoB*), and aminoglycosides (*aac(6′)-Ii*), respectively.

**Figure 3:**
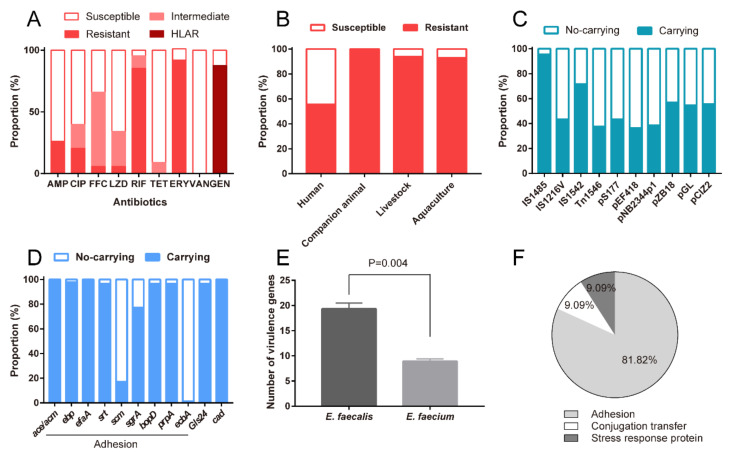
Risks of probiotic enterococcal isolates. (**A**) Resistance rates of probiotic enterococcal isolates to different antimicrobial drugs. (**B**) Resistance rates of probiotic enterococcal isolates used for different application targets. (**C**) Carrying rates of different mobile genetic elements. (**D**) Carrying rates of different virulence genes. (**E**) The number of virulence genes in *E. faecalis* and *E. faecium* isolates. (**F**) Different functions of virulence genes in these probiotic enterococcal isolates. Differences in E were statistically analyzed by Student’s *t*-test. *p* ≤ 0.05 was considered to be significant.

Subsequently, we assessed antimicrobial susceptibilities of the probiotic enterococcal isolates against nine kinds of antimicrobial agents. The results showed that all isolates (100%, 88/88) exhibited resistance to antimicrobial drugs, and 95.45% (84/88) of those conferred resistance to multi-antimicrobial drugs. As shown in Figure 3A, a large proportion of isolates exhibited resistance to AMP, CIP, RIF, and ERY with levels of 26.13% (22/88), 20.45% (18/88), 85.23% (75/88), and 92.05% (81/88), respectively. The enterococcal isolates were intermediate to FFC and LZD, with rates of 60.23% (53/88) and 26.14% (23/88). Notably, 87.5% (77/88) of these probiotic enterococcal isolates are highly resistant to Gen in our study (MICs of >500 μg/mL for gentamicin, known as high-level aminoglycoside resistance, HLAR). Besides, the rates of antimicrobial resistance of probiotic enterococcal isolates used for different targets varied. As shown in Figure 3B, the resistance rate of probiotic enterococci used for companion animals is the highest (100%), followed by 94% for livestock, 92.86% for aquaculture, and 55.56% for humans. Alarmingly, almost all the isolates (98.86%, 87/88) carried MGEs, which indicated that virulence determinants and antimicrobial resistance traits of enterococci may be plasmid-borne. Different MGE-carrying rates are shown in Figure 3C. Profiles of MGEs in probiotic enterococcal isolates used for different application targets are shown in Table S2.

### 3.5. Probiotic Enterococcal Isolates Carried VGs and Had Toxicity In Vitro

Through analyzing whole-genome data, we found that these enterococcal isolates have a series of VGs, including *acm*, *ebp*, *efaA*, *srt*, *scm*, *sgrA*, *bopD*, *prpA* and *ecbA* (Figure 2 and Figure 3D). In addition, more VGs were detected in all E. faecalis isolates (Table S3 and Figure 3E). As can be seen in Figure 3F, 81.82% of enterococcal isolates had VGs involved in biofilm formation and adhesion; 9.09% of enterococcal isolates had VGs involved in conjugation transfer, and 9.09% of enterococcal isolates had VGs involved in stress response protein. Profiles of VGs in probiotic enterococcal isolates used for different application targets are shown in Table S2.

**Figure 4:**
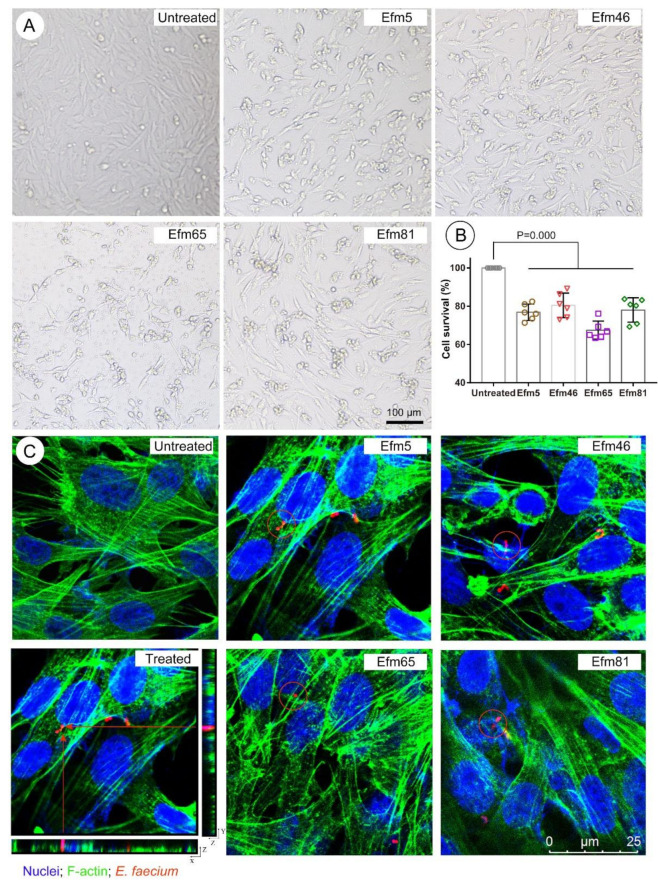
Toxicity evaluation of probiotic enterococcal isolates in vitro. (**A**) The morphology of human intestinal epithelial cells (HIEC cells) in different treated groups. Scale bar = 100 µm. (**B**) Rates of cell survival in different treated groups. (**C**) Probiotic enterococcal isolates internalization in human intestinal epithelial cells. F-actin was stained by GFP (green) and nuclei were counterstained with DAPI (blue). Enterococcal isolates were stained by rhodamine-phalloidin (red). Red arrows: Internalized bacteria in a 3D image. Red circles: Internalized bacteria. Scale bar = 25 µm. Differences in B were assessed by one-way ANOVA, and the values are presented as the mean ± SD. *p* ≤ 0.05 was considered to be significant.

As shown in Figure 4A, HIEC cells in the untreated group were polygonal in shape, with a round or elliptical shape; cells were atrophied and did not grow well in representative enterococcal isolates-treated groups. In Figure 4B, the cell survival of HIEC cells treated with four representative enterococcal isolates decreased significantly. Moreover, HIEC cells, co-incubated with the Efm65 isolate, had the lowest cell survival rate (Figure 4B), which means that the toxicity of Efm65 was the most. Moreover, these representative enterococcal isolates invaded human intestinal epithelial cells (Figure 4C), which means they could survive and persist in the cytosol of HIEC cells.

### 3.6. Representative Probiotic Enterococcal Isolates Had Toxicity In Vivo

**Figure 5:**
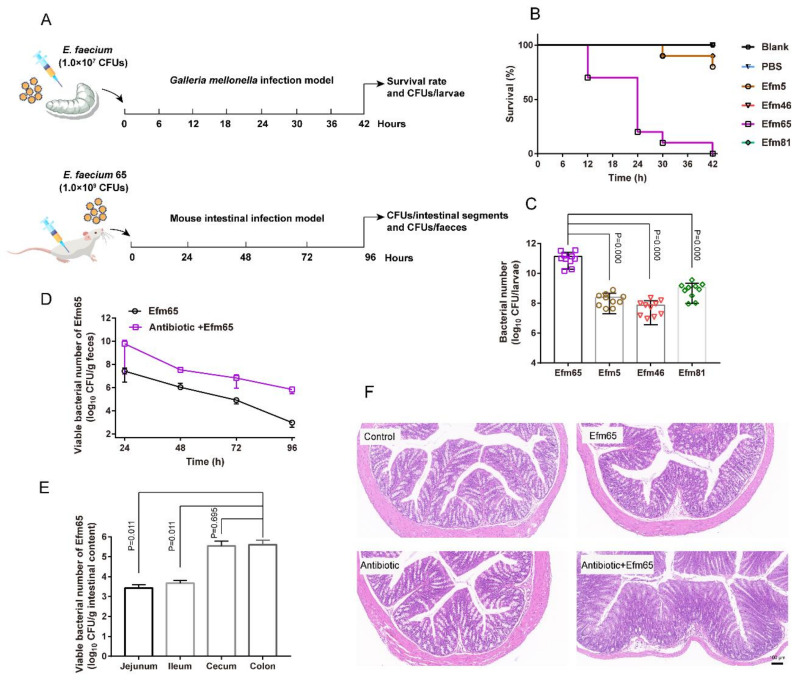
Toxicity evaluation of probiotic enterococcal isolates in vivo. (**A**) Scheme of the experimental protocol for the *G. mellonella* infection model and mouse intestinal infection model. (**B**) Survival rates of *G. mellonella* larva. Infected larvae (n = 10) with enterococcal isolates (10^7^ CFUs/larva). (**C**) The number of enterococci in *G. mellonella* posts 42 h infection. (**D**) Colonization of the Efm65 in the intestine of mice. (**E**) The number of the Efm65 colonized different mouse intestinal segments. (**F**) The morphology of mouse colonic tissues in different treated groups. Differences in C and E were statistically analyzed by one-way ANOVA. *p* ≤ 0.05 was considered to be significant.

We used a *Galleria mellonella* infection model to evaluate the toxicity of probiotic enterococcal isolates in vivo (Figure 5A). Melanization started on the larvae after the treatment of representative enterococcal isolates. The Efm65 isolate used for livestock made all the larvae completely melanized and dead within 42 h (Figure 5B). Our results showed that the number of Efm65 in *G. mellonella* post 42 h infection was significantly higher than other isolates (Figure 5C).

Therefore, we chose the isolate Efm65 for a mouse intestinal infection model to further evaluate the toxicity of probiotic enterococci in vivo (Figure 5A). Through the count of the bacterial population, we found that Efm65 could persist in the intestine of mice for 96 h; and the bacterial populations in feces in the antibiotic+Efm65 supplement group were higher than that in the Efm65 group (Figure 5D). As shown in Figure 5E, this demonstrated that Efm65 colonized the jejunum, ileum, cecum, and colon of mice, and the number of Efm65 in colonic contents was more than other intestinal segments. By histological analysis of mouse colonic tissues (Figure 5F), the results showed that Efm65 impaired the morphology of the colon and caused intestinal damage in the antibiotic+Efm65 supplement group. Collectively, the results illustrated that representative enterococcal isolates could proliferate rapidly in vivo and exert toxic effects on the host.

## 4. Discussion

Probiotics are popular on the food market for providing a health benefit to the host. However, there are rising risks of probiotics, especially for enterococci. Our study showed that these enterococci isolated from probiotic products exerted a harmful influence on the host by carrying toxicity, multiple antimicrobial resistance, and MGEs.

Firstly, probiotics have to survive during the transit through the upper gastrointestinal tract and then persist in the gut to provide effects for the host [24,25]. Our results showed that these representative enterococcal isolates survived steadily in simulated gastrointestinal juices. This means that they have the ability to survive under the harsh conditions of the gastrointestinal tract. Secondly, the colonization of probiotics in the intestinal epithelium is the prerequisite for their function. In this work, we demonstrated that all enterococcal isolates harbored VGs related to adhesion. For 85 *E. faecium* isolates, they harbored a lot of cell-surface-associated virulence genes, sex pheromone determinants (*cad*), and stress response proteins (*gls24*). Cell-surface-associated virulence genes could contribute to biofilm formation and adhesion to host tissues. In addition, *acm* [26], *efaA* [27], *ebp* [28], and *gls24* [29] are involved in the pathogenesis of endocarditis; *scm* has a high prevalence in clinically related *E. faecium* [30]; *sgrA* and *ecbA* are the marker for clinically associated *E. faecium* [31]; *bopD* contributes to prolonged mouse bacteremia [32]; *prpA* is proposed to contribute to colonization and infection, and its N-terminus is able to bind to fibrinogen, fibronectin and platelets [33]; *cad* contributes to facilitating conjugation, chemotactic for human neutrophils [34]. Therefore, the wide spectrum of VGs in these enterococcal isolates allows them to cause infection and disease.

Intestinal epithelial cells consist of a front-line barrier counteracting invasion in hosts [35,36]. This study demonstrated that these probiotic enterococcal isolates invaded human intestinal epithelial cells and caused cellular damage. The *G. mellonella* infection model has already been used to study the virulence of *E. faecium* [37,38,39]. Microbial virulence can be assessed by measuring the proliferation of the microorganism inside the larvae during infection [40]. In this work, representative enterococcal isolates proliferated rapidly and caused larvae death. In addition, we found that the morphology of intestinal epithelium was destroyed in probiotic-treated mice by the mouse intestinal infection model. Moreover, these virulent probiotic isolates and their toxins may contaminate water and foods through the food chain, and eventually endanger public health [41]. Taken together, such probiotic products harboring a toxigenic potential to produce virulence factors are not recommended for use.

One way for probiotics to protect intestinal health is to secrete antibacterial active molecules, and we could predict possible metabolites from the perspective of bacterial gene analysis [42]. However, no effective antibacterial active molecules were found in these probiotic enterococcal isolates. Worse still, we found that these enterococcal isolates had multi-antimicrobial resistance genes and phenotypes. Similar to this work, ERY, GEN, and TET resistance were found in enterococci species isolated from Turkish white cheese [43]. As we all know, aminoglycosides are important in the clinical treatment of enterococcal infections [44,45]. Nevertheless, most of these probiotic enterococcal isolates were highly resistant to GEN in our study. Therefore, the application of HLAR-enterococcal isolates in probiotic products poses a threat to public health. Moreover, almost all isolates harbored MGEs, which indicated that VGs and ARGs of enterococci may be plasmid-borne. The gastrointestinal system is a hot spot for genetic exchange. Experimental evidence has shown that antimicrobial resistance and virulence determinants are easily transferred between enterococci in the intestinal tract [46]. Therefore, these probiotic enterococcal isolates will be a reservoir for the exchanging and transmission of antimicrobial resistance and virulence determinants. The application of enterococci in probiotic products will be a great threat to the host and public health.

## 5. Conclusions

Altogether, our study shows that these probiotic enterococcal isolates not only have serious antimicrobial resistance but also proliferate rapidly in the host and provided toxic effects. The presence of MGEs in enterococcal isolates indicate that these microorganisms may transfer harmful factors into the environment during their growth. Therefore, greater attention should be paid to whether enterococci are applicable in probiotic products, and this has become a global issue that urgently needs to be solved.

## Supplementary Materials

The following are available online at https://www.mdpi.com/article/10.3390/foods10112846/s1, Figure S1: Number of enterococcal isolates used for different application targets, Figure S2: Representative probiotic enterococcal isolates survived steadily under simulated gastric and intestinal juices, Table S1: Information of probiotic enterococcal isolates used for different application targets, Table S2: Profiles of mobile genetic elements and virulence genes in probiotic enterococcal isolates used for different application targets, Table S3: Virulence genes of probiotic *E. faecalis* isolates.

## References

[1] Hill C., Guarner F., Reid G., Gibson G.R., Merenstein D.J., Pot B., Morelli L. (2014). Expert consensus document. The international scientific association for probiotics and prebiotics consensus statement on the scope and appropriate use of the term probiotic. Nat. Rev. Gastroenterol. Hepatol..

[2] Kim B., Wang Y.C., Hespen C.W., Espinosa J., Salje J., Rangan K.J., Oren D.A., Kang J.Y., Pedicord V.A., Hang H.C. (2019). *Enterococcus faecium* secreted antigen A generates muropeptides to enhance host immunity and limit bacterial pathogenesis. eLife.

[3] Ohland C.L., Macnaughton W.K. (2010). Probiotic bacteria and intestinal epithelial barrier function. Am. J. Physiol. Gastrointest. Liver Physiol..

[4] Tsai C.C., Hsih H.Y., Chiu H.H., Lai Y.Y., Liu J.H., Yu B., Tsen H.Y. (2005). Antagonistic activity against *Salmonella* infection in vitro and in vivo for two *Lactobacillus* strains from swine and poultry. Int. J. Food Microbiol..

[5] Piewngam P., Zheng Y., Nguyen T.H., Dickey S.W., Joo H.S., Villaruz A.E., Glose K.A., Fisher E.L., Hunt R.L., Li B. (2018). Pathogen elimination by probiotic *Bacillus* via signalling interference. Nature.

[6] WHO (2014). Antimicrobial Resistance: Global Report on Surveillance.

[7] Ben Braiek O., Smaoui S. (2019). Enterococci: Between emerging pathogens and potential probiotics. BioMed Res. Int..

[8] Suez J., Zmora N., Zilberman-Schapira G., Mor U., Dori-Bachash M., Bashiardes S., Zur M., Regev-Lehavi D., Ben-Zeev Brik R., Federici S. (2018). Post-antibiotic gut mucosal microbiome reconstitution is impaired by probiotics and improved by autologous FMT. Cell.

[9] Yelin I., Flett K.B., Merakou C., Mehrotra P., Stam J., Snesrud E., Hinkle M., Lesho E., McGann P., McAdam A.J. (2019). Genomic and epidemiological evidence of bacterial transmission from probiotic capsule to blood in ICU patients. Nat. Med..

[10] Gueimonde M., Sánchez B., de Los Reyes-Gavilán C.G., Margolles A. (2013). Antibiotic resistance in probiotic bacteria. Front. Microbiol..

[11] Cui Y., Martlbauer E., Dietrich R., Luo H., Ding S., Zhu K. (2019). Multifaceted toxin profile, an approach toward a better understanding of probiotic *Bacillus Cereus*. Crit. Rev. Toxicol..

[12] Cui Y., Wang S., Ding S., Shen J., Zhu K. (2020). Toxins and mobile antimicrobial resistance genes in *Bacillus* probiotics constitute a potential risk for One Health. J. Hazard. Mater..

[13] Fu S., Yang Q., He F., Lan R., Hao J., Ni P., Liu Y., Li R. (2020). National safety survey of animal-use commercial probiotics and its spillover effects from farm to human: An emerging threat to public health. Clin. Infect. Dis..

[14] Arias C.A., Murray B.E. (2012). The rise of the *Enterococcus*: Beyond vancomycin resistance. Nat. Rev. Microbiol..

[15] Sanders M.E., Akkermans L.M., Haller D., Hammerman C., Heimbach J., Hörmannsperger G., Huys G., Levy D.D., Lutgendorff F., Mack D. (2010). Safety assessment of probiotics for human use. Gut Microbes.

[16] Hanchi H., Mottawea W., Sebei K., Hammami R. (2018). The genus *Enterococcus*: Between probiotic potential and safety concerns-an update. Front. Microbiol..

[17] Wang X., Yang Y., Huycke M.M. (2020). Risks associated with enterococci as probiotics. Food Res. Int..

[18] Mater D.D., Langella P., Corthier G., Flores M.J. (2008). A probiotic *Lactobacillus* strain can acquire vancomycin resistance during digestive transit in mice. J. Microbiol. Biotechnol..

[19] Authority E.F.S. (2007). Introduction of a Qualified Presumption of Safety (QPS) approach for assessment of selected microorganisms referred to EFSA—Opinion of the Scientific Committee. EFSA J..

[20] Fang S.B. (2020). Enterococci and food safety—Are all probiotics beneficial?. Pediatr. Neonatol..

[21] Hempel S., Newberry S., Ruelaz A., Wang Z., Miles J.N., Suttorp M.J., Johnsen B., Shanman R., Slusser W., Fu N. (2011). Safety of probiotics used to reduce risk and prevent or treat disease. Evid. Rep. Technol. Assess..

[22] Ludwig W. (2007). Nucleic acid techniques in bacterial systematics and identification. Int. J. Food Microbiol..

[23] Zankari E., Hasman H., Cosentino S., Vestergaard M., Rasmussen S., Lund O., Aarestrup F.M., Larsen M.V. (2012). Identification of acquired antimicrobial resistance genes. J. Antimicrob. Chemother..

[24] Holzapfel W.H., Haberer P., Snel J., Schillinger U., in’t Veld J.H.H. (1998). Overview of gut flora and probiotics. Int. J. Food Microbiol..

[25] Chou L.S., Weimer B. (1999). Isolation and characterization of acid- and bile-tolerant isolates from strains of *Lactobacillus acidophilus*. J. Dairy Sci..

[26] Nallapareddy S.R., Singh K.V., Murray B.E. (2008). Contribution of the collagen adhesin Acm to pathogenesis of *Enterococcus faecium* in experimental endocarditis. Infect. Immun..

[27] Low Y.L., Jakubovics N.S., Flatman J.C., Jenkinson H.F., Smith A.W. (2003). Manganese-dependent regulation of the endocarditis-associated virulence factor EfaA of *Enterococcus faecalis*. J. Med. Microbiol..

[28] Singh K.V., Nallapareddy S.R., Murray B.E. (2007). Importance of the *ebp* (endocarditis- and biofilm-associated pilus) locus in the pathogenesis of *Enterococcus faecalis* ascending urinary tract infection. J. Infect. Dis..

[29] Nannini E.C., Teng F., Singh K.V., Murray B.E. (2005). Decreased virulence of a *gls24* mutant of *Enterococcus faecalis* OG1RF in an experimental endocarditis model. Infect. Immun..

[30] Yang J., Jiang Y., Guo L., Ye L., Ma Y., Luo Y. (2016). Prevalence of diverse clones of vancomycin-resistant *Enterococcus faecium* ST78 in a Chinese hospital. Microb. Drug Resist..

[31] Hendrickx A.P.A., van Wamel W.J.B., Posthuma G., Bonten M.J.M., Willems R.J.L. (2007). Five genes encoding surface-exposed LPXTG proteins are enriched in hospital-adapted *Enterococcus faecium* clonal complex 17 isolates. J. Bacteriol..

[32] Hufnagel M., Koch S., Creti R., Baldassarri L., Huebner J. (2004). A putative sugar-binding transcriptional regulator in a novel gene locus in *Enterococcus faecalis* contributes to production of biofilm and prolonged bacteremia in mice. J. Infect. Dis..

[33] Guzmán Prieto A.M., Urbanus R.T., Zhang X., Bierschenk D., Koekman C.A., van Luit-Asbroek M., Ouwerkerk J.P., Pape M., Paganelli F.L., Wobser D. (2015). The N-terminal domain of the thermo-regulated surface protein *PrpA* of *Enterococcus faecium* binds to fibrinogen, fibronectin and platelets. Sci. Rep..

[34] Bhardwaj A., Malik R.K., Chauhan P. (2008). Functional and safety aspects of enterococci in dairy foods. Indian J. Microbiol..

[35] König J., Wells J., Cani P.D., García-Ródenas C.L., MacDonald T., Mercenier A., Whyte J., Troost F., Brummer R.J. (2016). Human intestinal barrier function in health and disease. Clin. Transl. Gastroenterol..

[36] Ramanan D., Cadwell K. (2016). Intrinsic defense mechanisms of the intestinal eithelium. Cell Host Microbe.

[37] Chibebe Junior J., Fuchs B.B., Sabino C.P., Junqueira J.C., Jorge A.O.C., Ribeiro M.S., Gilmore M.S., Rice L.B., Tegos G.P., Hamblin M.R. (2013). Photodynamic and antibiotic therapy impair the pathogenesis of *Enterococcus faecium* in a whole animal insect model. PLoS ONE.

[38] Lebreton F., Le Bras F., Reffuveille F., Ladjouzi R., Giard J.C., Leclercq R., Giard J.C., Leclercq R., Cattoir V. (2011). *Galleria mellonella* as a model for studying *Enterococcus faecium* host persistence. J. Mol. Microbiol. Biotechnol..

[39] Lebreton F., van Schaik W., Sanguinetti M., Posteraro B., Torelli R., Le Bras F., Verneuil N., Zhang X., Giard J.C., Dhalluin A. (2012). AsrR is an oxidative stress sensing regulator modulating *Enterococcus faecium* opportunistic traits, antimicrobial resistance, and pathogenicity. PLoS Pathog..

[40] Tsai C.J., Loh J.M., Proft T. (2016). *Galleria mellonella* infection models for the study of bacterial diseases and for antimicrobial drug testing. Virulence.

[41] Stenfors Arnesen L.P., Fagerlund A., Granum P.E. (2008). From soil to gut: *Bacillus cereus* and its food poisoning toxins. FEMS Microbiol. Rev..

[42] Crits-Christoph A., Diamond S., Butterfield C.N., Thomas B.C., Banfield J.F. (2018). Novel soil bacteria possess diverse genes for secondary metabolite biosynthesis. Nature.

[43] Çitak S., Yucel N., Orhan S. (2004). Antibiotic resistance and incidence of *Enterococcus* species in Turkish white cheese. Int. J. Dairy Technol..

[44] Geraci J.E., Martin W.J. (1954). Antibiotic therapy of bacterial endocarditis. VI. Subacute enterococcal endocarditis; clinical, pathologic and therapeutic consideration of 33 cases. Circulation.

[45] Moellering R.C., Weinberg A.N. (1971). Studies on antibiotic syngerism against enterococci. II. Effect of various antibiotics on the uptake of 14 C-labeled streptomycin by enterococci. J. Clin. Investig..

[46] Coburn P.S., Baghdayan A.S., Dolan G.T., Shankar N. (2007). Horizontal transfer of virulence genes encoded on the *Enterococcus faecalis* pathogenicity island. Mol. Microbiol..

