# Supplementary material for: Emerging Risks in Food: Probiotic Enterococci Pose a Threat to Public Health through the Food Chain
Source: Foods. 2021 Nov 18;10(11):2846. doi: 10.3390/foods10112846 (PMC8623795; doi:10.3390/foods10112846)
Supplement: Supplementary file 1 [file foods-10-02846-s001.zip › Table S1 Information of probiotic enterococcal isolates used for different application targets.pdf]

Table S1. Information of probiotic enterococcal isolates used for different application targets

**Code of product**	**Origin**	**Name**	**Species**	**Amount per serving**	**Application targets**
1	Beijing	Efm1	*E. faecium*	Unknown	Human
2	Beijing	Efm2	*E. faecium*	Unknown	Human
3	Switzerland	Efm3	*E. faecium*	Unknown	Human
4	Japan	Efm4	*E. faecium*	2×10^5-4×10^8 CFUs per tablet	Human
5	Taiwan	Efm5	*E. faecium*	Unknown	Human
6	Jiangsu	Efm6	*E. faecium*	Unknown	Companion animal
7	Jiangsu	Efm7	*E. faecium*	1.0×10^{10} CFU/g	Companion animal
8	Japan	Efm8	*E. faecium*	300 billion/bag	Companion animal
9	Taiwan	Efm9	*E. faecium*	≥100 billion/g	Livestock
10	Taiwan	Efm10	*E. faecium*	≥2 billion/g	Livestock
11	Henan	Efm11	*E. faecium*	≥5 billion/g	Aquaculture
11	Henan	Efm12	*E. faecium*	≥5 billion/g	Livestock
12	Shandong	Efm13	*E. faecium*	≥4.5×10^{10} CFU/g	Livestock
13	Henan	Efm14	*E. faecium*	Unknown	Livestock
14	Jiangsu	Efm15	*E. faecium*	≥1 billion/g	Livestock
15	Liaoning	Efm16	*E. faecium*	Unknown	Livestock
16	Jiangsu	Efm17	*E. faecium*	Unknown	Livestock
17	Liaoning	Efm18	*E. faecium*	≥10 billion/g	Livestock
18	Hebei	Efm19	*E. faecium*	3-10 billion/g	Livestock
19	Shandong	Efm20	*E. faecium*	3-10 billion/g	Livestock
20	Guangdong	Efm21	*E. faecium*	Unknown	Livestock
21	Hubei	Efm22	*E. faecium*	≥10 billion/g	Livestock
22	Jiangsu	Efm23	*E. faecium*	Unknown	Livestock

23	Henan	Efm24	*E. faecium*	≥500 million/g	Livestock
24	Shandong	Efm25	*E. faecium*	Unknown	Livestock
25	Jiangxi	Efm26	*E. faecium*	≥10 billion/g	Livestock
26	Jiangxi	Efm27	*E. faecium*	Unknown	Livestock
26	Jiangxi	Efm28	*E. faecium*	≥1×10^{10} CFU/g	Livestock
27	Beijing	Efm29	*E. faecium*	30 billion/g	Livestock
28	Henan	Efm30	*E. faecium*	≥10 billion/g	Livestock
29	Guangdong	Efm31	*E. faecium*	Unknown	Livestock
30	Jiangsu	Efm32	*E. faecium*	Unknown	Livestock
31	Henan	Efm33	*E. faecium*	≥1.6×10^{9} CFU/g	Livestock
31	Henan	Efm34	*E. faecium*	≥1.6×10^{9} CFU/g	Livestock
32	Jiangxi	Efm35	*E. faecium*	Unknown	Aquaculture
33	Hubei	Efm36	*E. faecium*	Unknown	Aquaculture
34	Beijing	Efm37	*E. faecium*	20 billion/g	Aquaculture
35	Guangdong	Efm38	*E. faecium*	Unknown	Aquaculture
36	Hubei	Efm39	*E. faecium*	Unknown	Aquaculture
37	Beijing	Efm40	*E. faecium*	≥7.5×10^{7} CFU/g	Companion animal
38	Beijing	Efm41	*E. faecium*	≥1×10^{7} CFU/g	Companion animal
39	Jilin	Efm42	*E. faecium*	≥1.0×10^{8} CFU/g	Livestock
40	Jilin	Efm43	*E. faecium*	≥1.0×10^{8} CFU/g	Livestock
41	USA	Efm44	*E. faecium*	≥2×10^{7} CFU/g	Companion animal
42	Hebei	Efm45	*E. faecium*	≥1×10^{9} CFU/g	Companion animal
42	Hebei	Efm46	*E. faecium*	≥1×10^{9} CFU/g	Companion animal
43	Henan	Efm47	*E. faecium*	≥2×10^{10} CFU/g	Companion animal

44	UK	Efm48	*E. faecium*	≥2×10^{8} CFU/g	Companion animal
44	UK	Efm49	*E. faecium*	≥2×10^{8} CFU/g	Companion animal
45	USA	Efm50	*E. faecium*	≥2.5×10^{11} CFU/g	Companion animal
46	Hunan	Efm51	*E. faecium*	≥4.2×10^{8} CFU/g	Companion animal
46	Hunan	Efm52	*E. faecium*	≥4.2×10^{8} CFU/g	Companion animal
47	Hunan	Efm53	*E. faecium*	≥1.8×10^{8} CFU/g	Companion animal
48	Jiangxi	Efm54	*E. faecium*	≥1.8×10^{10} CFU/g	Aquaculture
49	Jiangxi	Efm55	*E. faecium*	≥4.5×10^{9} CFU/g	Livestock
49	Jiangxi	Efm56	*E. faecium*	≥4.5×10^{9} CFU/g	Livestock
50	Shandong	Efm57	*E. faecium*	≥1×10^{10} CFU/g	Livestock
51	Henan	Efm58	*E. faecium*	≥1×10^{6} CFU/g	Livestock
51	Henan	Efm59	*E. faecium*	≥1×10^{6} CFU/g	Livestock
52	Henan	Efm60	*E. faecium*	≥1×10^{6} CFU/g	Livestock
52	Henan	Efm61	*E. faecium*	≥1×10^{6} CFU/g	Livestock
52	Henan	Efm62	*E. faecium*	≥1×10^{6} CFU/g	Livestock
53	Henan	Efm63	*E. faecium*	100 billion/g	Livestock
53	Henan	Efm64	*E. faecium*	100 billion/g	Livestock
54	Henan	Efm65	*E. faecium*	Unknown	Aquaculture
54	Henan	Efm66	*E. faecium*	Unknown	Aquaculture
55	Henan	Efm67	*E. faecium*	Unknown	Livestock
55	Henan	Efm68	*E. faecium*	Unknown	Livestock
56	Henan	Efm69	*E. faecium*	Unknown	Aquaculture
57	Henan	Efm70	*E. faecium*	Unknown	Livestock
58	Henan	Efm71	*E. faecium*	Unknown	Aquaculture

58	Henan	Efm72	*E. faecium*	Unknown	Aquaculture
59	Henan	Efm73	*E. faecium*	≥10 billion/g	Livestock
59	Henan	Efm74	*E. faecium*	≥10 billion/g	Livestock
60	Henan	Efm75	*E. faecium*	≥10 billion/g	Livestock
60	Henan	Efm76	*E. faecium*	≥10 billion/g	Livestock
61	Henan	Efm77	*E. faecium*	≥10 billion/g	Livestock
62	Henan	Efm78	*E. faecium*	≥10 billion/g	Livestock
63	Jiangsu	Efm79	*E. faecium*	Unknown	Livestock
64	Henan	Efm80	*E. faecium*	Unknown	Aquaculture
65	Henan	Efm81	*E. faecium*	Unknown	Livestock
66	Henan	Efm82	*E. faecium*	Unknown	Aquaculture
67	Henan	Efm83	*E. faecium*	≥100 million/g	Human
68	Jiangsu	Efm84	*E. faecium*	Unknown	Livestock
69	Jiangsu	Efm85	*E. faecium*	Unknown	Livestock
70	Zhejiang	Efs1	*E. faecalis*	≥1×10^{6} CFU/g	Human
71	Japan	Efs2	*E. faecalis*	Unknown	Human
72	Shanghai	Efs3	*E. faecalis*	Unknown	Human

Note: The same code of product trade means that strains are isolated from the same probiotic product.
