# Supplementary material for: Emerging Risks in Food: Probiotic Enterococci Pose a Threat to Public Health through the Food Chain
Source: Foods. 2021 Nov 18;10(11):2846. doi: 10.3390/foods10112846 (PMC8623795; doi:10.3390/foods10112846)
Supplement: Supplementary file 1 [file foods-10-02846-s001.zip › Table S2 Profiles of mobile genetic elements and virulence genes in probiotic enterococcal isolates used for different application targets.pdf]

Table S2. Profiles of mobile genetic elements and virulence genes in probiotic enterococcal isolates used for different application targets

**Risk factors**	**Application targets**			
Mobile genetic elements	Human (9)	Companion animal (15)	Livestock (50)	Aquaculture (14)
IS1485	100% (9)	100% (15)	94% (47)	26% (13)
IS1216V	88.89% (8)	40% (6)	30% (15)	18% (9)
IS1542	55.56% (5)	93.33% (14)	80% (40)	8% (4)
Tn1546	66.67% (6)	33.33% (5)	26% (13)	18% (9)
pS177	88.89% (8)	40% (6)	25% (15)	18% (9)
pEF418	66.67% (6)	33.33% (5)	26% (13)	16% (8)
pNB2354p1	55.56% (5)	40% (6)	28% (14)	18% (9)
pNB18	55.56% (5)	66.67% (10)	62% (31)	8% (4)
pGL	22.22% (2)	60% (9)	68% (34)	6% (3)
pCIZ2	22.22% (2)	60% (9)	70% (35)	6% (3)
Virulence genes	Human (9)	Companion animal (15)	Livestock (50)	Aquaculture (14)
ace/acm	100% (9)	100% (15)	100% (50)	100% (14)
cad	100% (9)	100% (15)	100% (50)	100% (14)
ebp	100% (9)	93.33% (14)	98% (49)	100% (14)
efaA	100% (9)	100% (15)	100% (50)	100% (14)
srt	100% (9)	86.67% (13)	98% (49)	100% (14)
scm	44.44% (4)	33.33% (5)	12% (6)	0% (0)
sgrA	22.22% (2)	60% (9)	86% (43)	100% (14)
bopD	66.67% (6)	100% (15)	100% (50)	100% (14)
Gls24	77.78% (7)	93.33% (14)	100% (50)	100% (14)
mreC	66.67% (6)	100% (15)	100% (50)	100% (14)
prpA	66.67% (6)	100% (15)	100% (50)	100% (14)
