# Supplementary material for: Emerging Risks in Food: Probiotic Enterococci Pose a Threat to Public Health through the Food Chain
Source: Foods. 2021 Nov 18;10(11):2846. doi: 10.3390/foods10112846 (PMC8623795; doi:10.3390/foods10112846)
Supplement: Supplementary file 1 [file foods-10-02846-s001.zip › Table S3 Virulence genes of probiotic E. faecalis isolates.pdf]

Table S3. Virulence genes of probiotic *E. faecalis* isolates.

Virulence genes	*E. faecalis* 1	*E. faecalis* 2	*E. faecalis* 3
ace	+	+	+
cpd	+	+	+
cad	+	+	+
camE	+	+	+
ebp	+	+	+
efaA	+	+	+
ElrA	+	+	+
esp	-	+	+
fsr	+	+	+
gelE	+	+	+
hyl	+	+	+
srt	+	+	+
tpx	+	+	+
sprE	+	+	+
ccf	+	+	+
SigV	+	+	+
Npr	+	+	+
Gls24	+	+	+
ecbA	-	+	+
Cob1	+	+	+

+: indicating that this strain harbors the virulence gene; -: indicating that this strain doesn't harbor the virulence gene.
