# Supplementary figures and images for: Emerging Risks in Food: Probiotic Enterococci Pose a Threat to Public Health through the Food Chain
Source: Foods. 2021 Nov 18;10(11):2846. doi: 10.3390/foods10112846 (PMC8623795; doi:10.3390/foods10112846)

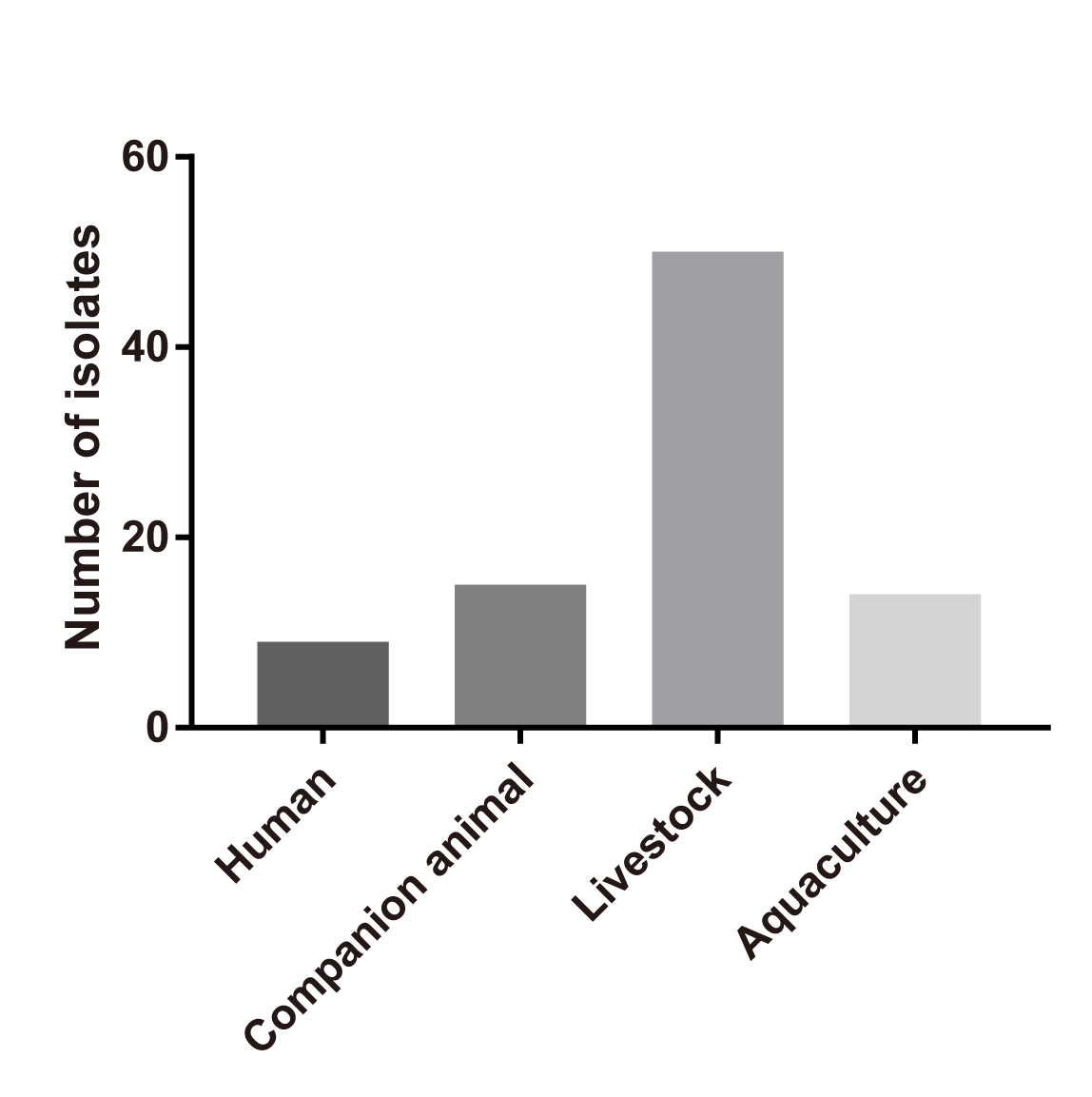

Supplement: Supplementary file 1 [file foods-10-02846-s001.zip › Figure S1Number of enterococcal isolates used for different application targets.tif]

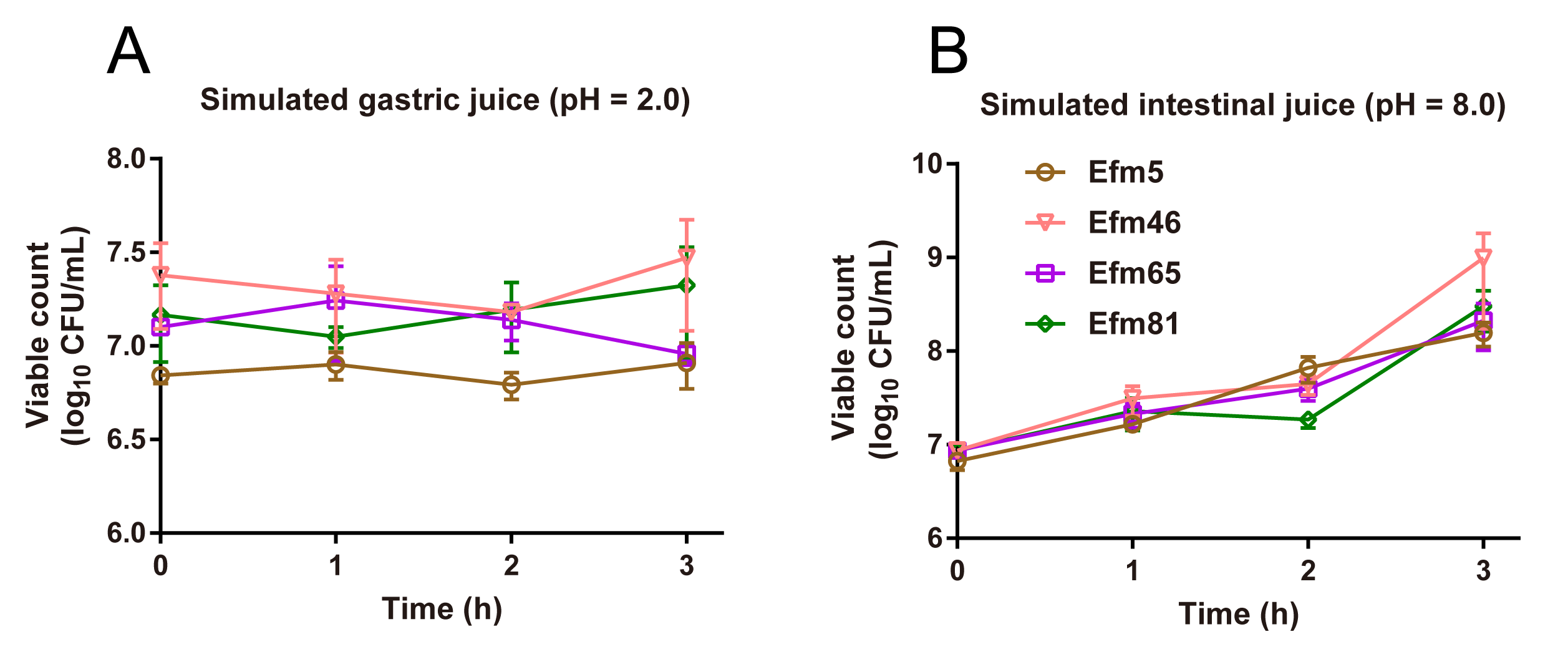

Supplement: Supplementary file 1 [file foods-10-02846-s001.zip › Figure S2 Representative probiotic enterococcal isolates survived steadily under simulated gastric and intestinal juices.tif]
